# Evolutionary History of HIV-1 Subtype B and CRF01_AE Transmission Clusters among Men Who Have Sex with Men (MSM) in Kuala Lumpur, Malaysia

**DOI:** 10.1371/journal.pone.0067286

**Published:** 2013-06-20

**Authors:** Kim Tien Ng, Lai Yee Ong, Sin How Lim, Yutaka Takebe, Adeeba Kamarulzaman, Kok Keng Tee

**Affiliations:** 1 Centre of Excellence for Research in AIDS (CERiA), Department of Medicine, Faculty of Medicine, University of Malaya, Kuala Lumpur, Malaysia; 2 AIDS Research Center, National Institute of Infectious Diseases, Toyama, Shinjuku-ku, Tokyo, Japan; Institut Pasteur of Shanghai, Chinese Academy of Sciences, China

## Abstract

HIV-1 epidemics among men who have sex with men (MSM) continue to expand in developed and developing countries. Although HIV infection in MSM is amongst the highest of the key affected populations in many countries in Southeast Asia, comprehensive molecular epidemiological study of HIV-1 among MSM remains inadequate in the region including in Malaysia. Here, we reported the phylodynamic profiles of HIV-1 genotypes circulating among MSM population in Kuala Lumpur, Malaysia. A total of n = 459 newly-diagnosed treatment-naïve consenting subjects were recruited between March 2006 and August 2012, of whom 87 (18.9%) were self-reported MSM. Transmitted drug resistance mutations were absent in these isolates. Cumulatively, phylogenetic reconstructions of the *pro-rt* gene (HXB2∶2253–3275) showed that HIV-1 subtype B and CRF01_AE were predominant and contributed to approximately 80% of the total HIV-1 infection among MSM. In addition to numerous unique transmission lineages within these genotypes, twelve monophyletic transmission clusters of different sizes (2–7 MSM sequences, supported by posterior probability value of 1) were identified in Malaysia. Bayesian coalescent analysis estimated that the divergence times for these clusters were mainly dated between 1995 and 2005 with four major transmission clusters radiating at least 12 years ago suggesting that active spread of multiple sub-epidemic clusters occurred during this period. The changes in effective population size of subtype B showed an exponential growth within 5 years between 1988 and 1993, while CRF01_AE lineage exhibited similar expansion between 1993 and 2003. Our study provides the first insight of the phylodynamic profile of HIV-1 subtype B and CRF01_AE circulating among MSM population in Kuala Lumpur, Malaysia, unravelling the importance of understanding transmission behaviours as well as evolutionary history of HIV-1 in assessing the risk of outbreak or epidemic expansion.

## Introduction

Human immunodeficiency virus type 1 (HIV-1) is an RNA virus that is known for its extreme genetic variability, owing to its high mutation rates, high viral turnovers and persistent nature of infections [1]. In addition to the 9 major subtypes (A - D, F - H, J and K) of the HIV-1 group M, at least 58 known circulating recombinant forms (CRFs) and various unique recombinant forms (URF) have been generated by extensive genetic recombination among HIV-1 subtypes.

HIV-1 infections among men who have sex with men (MSM) continue to expand worldwide [2]. In contrast to the low and decreasing HIV-1 prevalence among general adult population in most countries, MSM population continues to be disproportionately affected by HIV-1 infection, with MSM in Asia having approximately 19 times the odds of becoming infected [3]. Recent reports have shown the significant prevalence of HIV-1 infections among MSM in several Asian countries [4]–[6].

In Malaysia, a cumulative total of 94,841 cases of HIV-1 infections have been reported by the end of 2011, of which approximately 2.5% (2,406 cases) were associated with MSM (HIV/STI MOH, Malaysia). According to Kanter et al. [5], Malaysian MSM are commonly engaged in high-risk sexual behaviours and lack knowledge on the risks and transmission mode of HIV-1 [5], predisposing them to the continual exposure to and spread of HIV-1 infection. In a previous epidemiological study in 2003–2005 involving 184 HIV-infected subjects from various risk groups, it was shown that the MSM population was mainly infected with HIV-1 subtype B (45%), CRF01_AE (25%), CRF33_01B (19%) and other minor recombinant forms involving subtype B and CRF01_AE [7]. Despite the significant epidemiological impact attributed to HIV-1 subtype B and CRF01_AE in Malaysia and also elsewhere in the region, the pattern of transmission clusters, population dynamics and genetic history of these genotypes among the MSM population remains largely unknown. Through better understanding of transmission clusters, phylodynamics and genetic history of HIV-1 on a population level, effective preventive approaches can be designed to target factors associated with onward HIV-1 transmission [8], [9]. In the present study, based on contemporary HIV-1 sequence data generated from the antiretroviral resistance surveillance program, we investigated the phylodynamic profiles of HIV-1 subtype B and CRF01_AE lineages isolated from the MSM population in Malaysia, using a suite of phylogenetic tools that involve the maximum likelihood and Bayesian coalescence strategy.

## Materials and Methods

### Ethics Statement

The study was approved by the University Malaya Medical Centre (UMMC) Medical Ethics Committee. Standard, multilingual consent forms validated by the Medical Ethics Committee were used. Written consent was obtained from all study participants.

### Study Subjects and Specimen

A total of n = 459 consenting treatment-naïve HIV-1 positive subjects were recruited from UMMC in Kuala Lumpur, Malaysia between March 2006 and August 2012. Demographic information and transmission risk factors were obtained, and plasma specimens from these patients were serologically tested and confirmed to be HIV-1 positive.

### Viral RNA Extraction, *pro-rt* gene Amplification and Sequencing

In accordance with the manufacturer′s instruction, viral RNA was extracted from 140 µl of plasma samples using QIAmp Viral RNA Mini Kit (Qiagen, Valencia, California, USA), and was suspended in 50 µl elution buffer. The eluted RNA was reverse transcribed into cDNA with the SuperScript III reverse transcriptase (Invitrogen, USA). Nested-PCR was performed with various primer sets previously published [10]–[12], to amplify the *pro-rt* gene (HXB2∶2253–3275, 1022 bp) of HIV-1 by forming overlapping fragments. The 50 µl reaction mixture contained 20 µM of each primer, dNTPs (10 mM), 10X buffer with MgCl_2_, *Taq* polymerase and 2 µl of cDNA template. The PCR conditions were 5 minutes of initial denaturation at 95°C, followed by 35 cycles of denaturation (94°C for 30 seconds), annealing (50°C for 1 minute), extension (72°C for 1 minute 45 seconds) and final extension at 72°C for 10 minutes. The same reaction volume and cycling conditions were employed for the second PCR. The PCR products were visualized by gel electrophoresis, purified and sequenced by an ABI PRISM 3730XL DNA Analyzer (Applied Biosystems, USA). The nucleotide sequences were manually inspected and corrected prior to assembly. The sequences were then submitted to the BLAST program (http://blast.ncbi.nlm.nih.gov/) to check for possible contamination, followed by determination of possible recombinants using the Recombinant Identification Program (RIP) in the HIV database (http://www.hiv.lanl.gov) followed by similarity plot using SimPlot with window size of 300 bp and step size of 30 bp. These nucleotide sequences were codon aligned along with a comprehensive list of reference sequences retrieved from the HIV database. In order to determine the prevalence of transmitted drug resistance mutations among the antiretroviral-naïve MSM in Kuala Lumpur, drug resistance mutations conferring resistance to protease inhibitors (PIs), nucleoside reverse transcriptase inhibitors (NRTIs) and non-nucleoside reverse transcriptase inhibitors (NNRTIs) in the protease (PR) and reverse transcriptase (RT) genes (codons 1–99 and 1–253, respectively) were interpreted using the Stanford HIV Drug Resistance Database (http://hivdb.stanford.edu) and compared with the 2009 World Health Organization (WHO) guidelines for surveillance of transmitted drug resistance mutations [13].

### Phylodynamic Inference and Divergence Times of Transmission Clusters

Genetic subtype and potential transmission clusters from the time-stamped sequence dataset were first deduced by neighbour-joining tree reconstruction [14] using MEGA version 5.05 based on Kimura-2 parameter model [15]. The robustness of the transmission clusters were further tested by the more rigorous maximum likelihood inference implemented in PAUP version 4.0 [16] using gamma distribution with discrete gamma categories. The reliability of the branching orders was assessed by bootstrap analysis of 1000 replicates. The most appropriate nucleotide substitution model was determined using FindModel, a web implementation of Modeltest available at the HIV database (http://www.lanl.gov.com). In addition, Bayesian maximum clade credibility (MCC) trees were constructed separately using BEAST 1.7 [17] to determine the posterior probability values for each cluster in subtype B and CRF01_AE. In this study, identification of transmission clusters was based on recently reported guidelines, where a transmission cluster is characterized by the following criteria: (a) a cluster consisting of at least 2 isolates from different individuals of the same geographical (i.e country) origin [18], and (b) a phylogenetic clade supported by high bootstrap values (>90%) [19] and Bayesian posterior probability value of 1 at the tree node [19], [20].

To estimate the divergence times of the respective subtype B and CRF01_AE transmission clusters, the Bayesian coalescent-based relaxed molecular clock model was performed in BEAST 1.7 [17]. The uncorrelated lognormal model [21] nested in general time-reversible (GTR) nucleotide substitution model [22] with a proportion of invariant sites and four rate categories of gamma-distribution model of among site rate heterogeneity [23] was employed to estimate viral phylogenies, nucleotide substitution rates and to date the time of the most recent common ancestor (tMRCAs) for the respective subtype B and CRF01_AE transmission clusters. As for the coalescent priors, different parametric demographic models namely, constant population size, exponential and logistic growth as well as a nonparametric Bayesian skyline plot (BSP) was applied. The best fits coalescent model was chosen by means of a Bayes factor (BF), using marginal likelihoods, determined by Tracer version 1.5 (http://tree.bio.ed.ac.uk/software/tracer) [24]. The Markov chain Monte Carlo (MCMC) analysis was computed for 50 million states sampled every 10,000 states and output was assessed for convergence by means of effective sampling size (ESS) after a 10% burn-in using Tracer version 1.5. Since higher ESS value indicates lower standard errors, only traces with ESS of more than 200 were accepted. In order to infer the tMRCA for each transmission clusters with greater confidence [25] divergence time for subtype B′ of Thai origin (isolates 93CNRL42, 96M145, 96TH_NP1538, 96M081, 99TH_C1416, 99MMmSTD101, 00TH_C3198, 01CNHN24, 02CNHNsc11, 02CNHNsmx2 and 02CNHNsq4), which were thought to be descended from the ancestral subtype B lineages around 1980 to 1991 [26], [27], were co-estimated and checked for concordance with the current estimates.

### Sequences

Partial sequences of the HIV *pol* gene analyzed in this study have been deposited in GenBank under accession numbers KC181210– KC181243 and KC740499– KC7400551.

## Results

In the present study, a cohort of n = 459 treatment-naïve HIV-1 positive subjects were consented and recruited between March 2006 and August 2012, of whom 87 (18.9%) were MSM (80 homosexuals and 7 bisexuals), of different ethnicity, namely Chinese (64.4%), Malay (25.3%), Indian (6.9%) and other ethnic groups (3.4%). All HIV-1 *pro-rt* gene (HXB2∶2253–3275) were sequenced and subjected to genotypic drug resistance testing based on the 2009 WHO guidelines [13], which indicated the absence of transmitted drug resistance mutations among treatment-naïve MSM in Kuala Lumpur, Malaysia, in line with evidence from a previous study [11].

**Figure 1:**
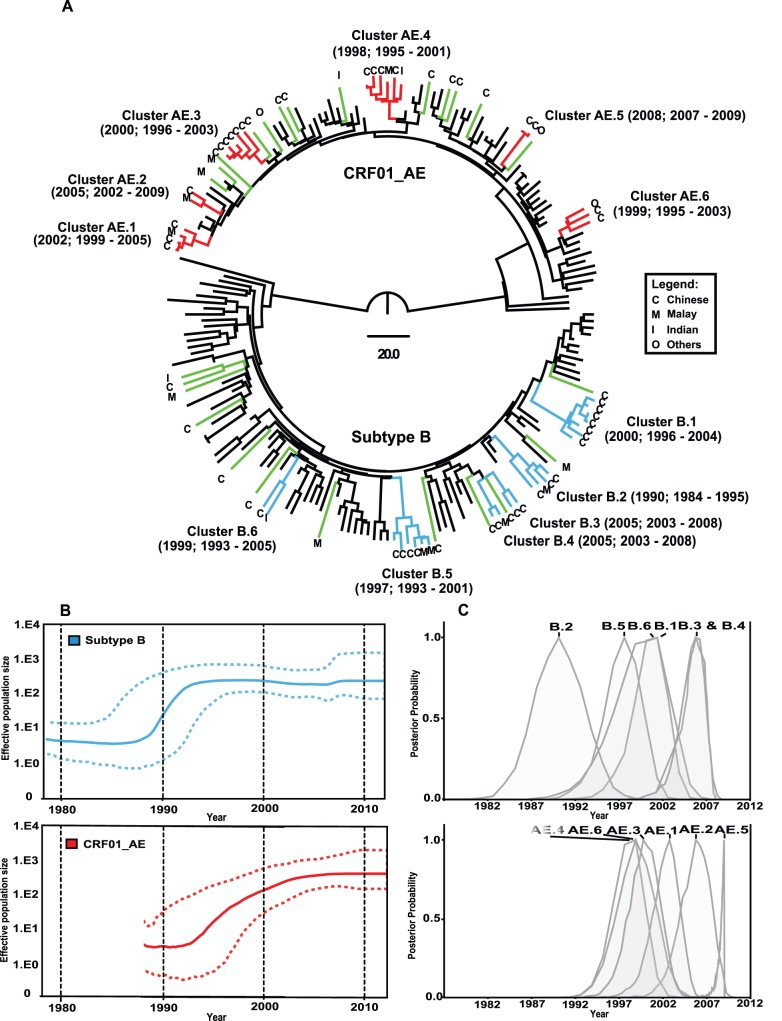
Phylodynamic profile of HIV-1 subtype B and CRF01_AE among men who have sex with men (MSM) in Kuala Lumpur, Malaysia. The Malaysian MSM sequences (35 subtype B and 35 CRF01_AE) along with respective reference sequences were analyzed separately. For illustration purposes, all 70 MSM sequences were presented in the same maximum clade credibility (MCC) tree. (**A**) Bayesian’s MCC tree of 1022 bp *pro-rt* gene (HXB2∶2253–3275) of 70 Malaysian MSM sequence data collected between March 2006 and August 2012 and reference sequences (black branches) is shown. The Bayesian coalescent-based relaxed molecular clock model was performed in BEAST 1.7, with uncorrelated lognormal model nested in general time-reversible (GTR) nucleotide substitution model and a proportion of invariant sites. The Markov chain Monte Carlo (MCMC) analysis was computed for 50 million states sampled every 10,000 states and output was assessed for convergence by means of effective sampling size (ESS) after a 10% burn-in. Transmission clusters are defined based on strong statistical supports generated at the internal nodes of the maximum likelihood and MCC tree reconstructions (bootstrap values of more than 90% and posterior probability of 1, respectively). A total of 12 monophyletic transmission clusters of different sizes (2–7 sequences) were determined, of which 6 clusters (B.1– B.6) were found within the subtype B lineages (blue branches) and another 6 clusters (AE.1– AE.6) were identified within the CRF01_AE lineages (red branches). The mean tMRCA and 95% highest posterior distribution (HPD) for each cluster are indicated in parentheses. In addition, 25 single unique lineages (green branches) involving subtype B and CRF01_AE are indicated in the phylogenetic analysis. The scale bar indicates the time in years and the alphabet at the tip of each branch represents the ethnicity of the subject, namely Malay (M), Chinese (C), Indian (I) and others (O). (**B**) Bayesian skyline plots (BSPs) generated from 35 HIV-1 subtype B and 35 CRF01_AE heterochronously sampled *pro-rt* gene from the MSM population in Kuala Lumpur. The origin and changes in effective population size through time for subtype B and CRF01_AE in the country were estimated. The 95% HPD of the effective population size is indicated in dashed lines. (**C**) Relative posterior probability distribution of the tMRCAs for the respective subtype B and CRF01_AE transmission clusters.

Phylogenetic reconstruction revealed that the main HIV-1 genotypes circulating among MSM in Kuala Lumpur were CRF01_AE (35/87, 40.2%) and subtype B (35/87, 40.2%). Of the 35 subtype B sequences, 27 (77.1%) were subtype B of western origin and the remaining were subtype B′ of Thai origin. Phylogenetic inference was also used to determine the structure of transmission events at the population level. Based on the strong statistical support generated at the internal tree nodes of maximum likelihood and Bayesian’s maximum clade credibility (MCC) tree analysis (bootstrap values of more than 90% and posterior probability of 1, respectively), 45 Malaysian MSM infected with subtype B and CRF01_AE formed twelve transmission clusters of different sizes (2–7 sequences), six monophyletic transmission clusters were observed within subtype B (denoted cluster B.1 to B.6) and CRF01_AE (cluster AE.1 to AE.6) lineages, respectively **(** **Fig. 1A** **)**. In addition, 25 single unique lineages (12 and 13 isolates within subtype B and CRF01_AE, respectively) were identified. Chi-square analysis indicate that clustering pattern have no obvious correlation with ethnicity (*P*  = .2146), age (*P*  = .188) and viral load (*P*  = .542).

Analyses of these *pro-rt* sequences under a relaxed clock (uncorrelated lognormal) and constant coalescent model revealed a mean evolutionary rate of 1.9–2.0 (1.7–2.2)×10^−3^ substitutions/site/year, consistent with previously published estimates [20], [28]. Based on these evolutionary parameters, the time to the most recent common ancestor (tMRCA) of each transmission cluster was estimated. Transmission clusters of subtype B mainly emerged around early 1990s to mid 2000s with the oldest cluster most likely emerging as early as 1984, while CRF01_AE transmission clusters diverged later, from mid 1990s to late 2000s **(** **Fig. 1A** **)**. In addition, the tMRCA of reference subtype B′ was estimated at 1986 (95% credible region: 1984–1989), in congruence with the findings from other studies [26], [27], [29].

To illustrate the past population dynamics of HIV-1 circulating among the Malaysian MSM, temporal changes in effective population size over the last three decades for subtype B and CRF01_AE were estimated **(** **Fig. 1B** **)**. Bayesian skyline plot for subtype B lineage indicated the initial growth phase started in early 1980s, followed by an exponential growth between 1988 and 1992, and reached its peak from 1995 onwards. For CRF01_AE lineage, the initial growth phase radiated in the late 1980s, and later undergone regular increase around 1993 to 2003, and reached sustainable growth thereafter. Taken together, the present study indicates that the divergence times for these sequence clusters were dated mostly between 1995 and 2005.

To estimate the year of transmission events within cluster, each heterochronously sampled HIV *pro-rt* gene (HXB2∶2253–3275) in the time-scaled phylogeny represents a different patient, and distance between two adjacent tMRCAs denotes the upper boundary of the time between 2 transmission events. Estimation of the year for each node in the major transmission clusters (Cluster B.1, B.5, AE.3, and AE.4) revealed that the median interval between transmission events within these clusters were relatively short: 1.6 years (1.1–5.3) in Cluster B.1, 3.4 years (1.6–4.3) in Cluster B.5, 2.2 years (1.6–2.7) in Cluster AE.3 and 3.3 years (1.5–4.2) in Cluster AE.4. On average, transmission clusters formed within CRF01_AE showed shorter transmission intervals (2.4 years; 1.6–3.3 years) compared to those within subtype B (3.8 years; 1.6–6.5 years).

**Figure 2:**
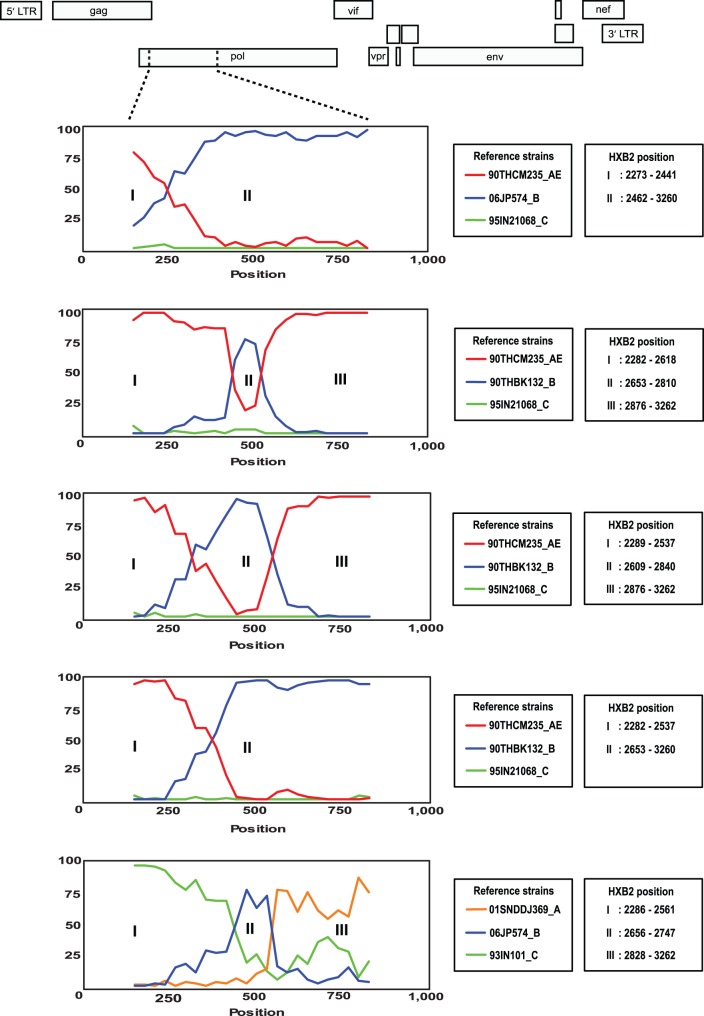
Mosaic structures of unique recombinant forms (URF) among men who have sex with men (MSM). The closely related putative parental strains for each URF were determined by similarity plot. The bootscan plots of *pro-rt* gene (HXB2∶2253–3275) with window size of 300 bp and step size of 30 bp illustrate the relationship of Malaysian URF to the reference strains of HIV-1 subtype B of western origin, CRF01_AE, subtype C and subtype A. The recombination breakpoint(s) in relation to HXB2 coordinates are indicated in the inset.

Apart from HIV-1 subtype B and CRF01_AE, MSM in Kuala Lumpur, Malaysia were also infected with HIV-1 CRF33_01B (10/87, 11.5%), CRF51_01B (1/87, 1.1%), and subtype C (1/87, 1.1%). In addition, CRF01_AE/B and subtype A/B/C unique recombinant forms were estimated at 4.6% (4/87) and 1.1% (1/87), respectively, with the mosaic structure determined using RIP tool and bootscan analysis **(** **Fig. 2** **)**.

## Discussion

Although HIV-1 transmission is increasing among the MSM population, a comprehensive view of the HIV-1 genomic variability, growth and temporal dynamics among MSM remains inadequate particularly in Southeast Asia. To our knowledge, this is the first molecular epidemiological study in Kuala Lumpur, Malaysia that investigates the transmission pattern and evolutionary history of HIV-1 subtype B and CRF01_AE circulating among the MSM population based on the contemporary time-stamped sequence data collected between 2006 and 2012. Here, we highlight three possible key factors that might have contributed to the pattern of HIV-1 infections among MSM in Kuala Lumpur, namely the establishment of transmission clusters, dissemination of HIV-1 through MSM network and lastly, the emergence of HIV-1 variants.

The HIV-1 *pol* gene generated from antiretroviral resistance surveillance programs has been proven useful and informative in assessing and defining transmission clusters within a population of interest [30]–[32]. By definition, transmission clusters are phylogenetic clusters consisting of 2 or more nucleotide sequences from different individuals that are supported by strong statistical inference, namely bootstrap value of more than 90% and Bayesian posterior probability of 1. Based on these criteria, substantial clustering (51.7%; 45/87) was observed, indicating that the majority of HIV-1 infections among MSM in Kuala Lumpur were linked to a cluster that may be associated with local and/or foreign MSM networks. On the other hand, the 25 unique transmission lineages observed in present study could be due to incomplete sampling. Bayesian coalescent analysis estimated that the divergence times for most of the transmission clusters originated between 1995 and 2005, with the major transmission clusters (Cluster B.1, B.5, AE.3 and AE.4) radiating at least 12 years ago. The relatively short median interval between transmission events within the major clusters indicate an ongoing active HIV-1 transmission and suggest possible epidemic expansion due to high-risk sexual behaviour among the MSM population [5], [33], [34]. The identification of viral transmission clusters coupled with the estimated divergence time suggest that HIV-1 subtype B and CRF01_AE lineages were introduced into the MSM population in Kuala Lumpur through multiple sub-epidemics, most of which occurred between 1995 and 2005 (58%). Interestingly, other studies conducted in Western Europe [20], [33], [34] reported similar findings during the same period, indicating that the emergence of transmission clusters was likely caused by the increasing exposure to high-risk behaviours among MSM. In addition, Brenner et al [35] had demonstrated the positive correlation between clusters size, transmission events and expansion rate, underscoring the importance of understanding these transmission clusters during assessment of ongoing outbreak or expanding epidemic. However, in the present study, such correlation was not observed, possibly due to the smaller sample size of the study. In order to have a more representative finding, an in-depth sampling method spanning a longer recruitment period should be employed.

The first cases of AIDS were documented in Malaysia in 1986 [35]. Of note, the tMRCA of the oldest subtype B cluster estimated in the present study was dated as early as 1984, corroborating with epidemiological data that HIV-1 might have been introduced into the Malaysian MSM population around the mid 1980s. Genetic data also demonstrated the evolutionary history of HIV-1 subtype B and CRF01_AE lineages spanning a period of almost three decades. Collectively, the Bayesian skyline plot showed an epidemic expansion from late 1980s to mid-1990s. The changes in effective population size of subtype B showed an approximate 10-fold exponential growth within 5 years (1988 to 1993), while CRF01_AE lineage exhibited an estimated 10-fold sustained growth in 10 years (1993 to 2003).

In line with a previous study [7], subtype B and CRF01_AE remain as the dominant co-circulating subtypes, contributing to approximately 80% of the total HIV-1 infection among MSM in Kuala Lumpur. Similar findings were reported in neighbouring country Singapore [36]. In the present study, a number of recombinant involving subtype A/B/C and CRF01_AE/B were also observed. Interestingly, the subtype B region of these URFs in Malaysian MSM population is of western B origin, distinguishing them from the URFs recently described among people who inject drugs that involved subtype B′ of Thai origin [7], [29]. The emergence of these variants indicates an ongoing active intersubtype recombination between the predominant genotypes that may complicate disease management. In the present study, the absence of genotypic drug resistance mutations among treatment-naïve HIV-1-infected MSM in Kuala Lumpur suggests that the transmission of drug-resistant variants remains low, despite the active rollout of highly active antiretroviral therapy (HAART) in Malaysia since early 2000s. A more representative sample size involving recent seroconverters from other regions in Malaysia is required to precisely evaluate the transmission of drug resistance mutations in the country. Importantly, this finding also eliminates one possible bias in assigning monophyletic transmission clusters attributed to the effects of convergence evolution due to drug therapy-associated selection pressure [18], [20], [32].

From the public health perspective, sexual networks within the MSM population could serve as an important force of continual HIV-1 dissemination, thus it functions as the key entry points for the delivery of intervention strategies. In 2011, Kanter et al. [5] reported significant association between high risk sexual behaviour and little knowledge on HIV transmission with increased vulnerability of Malaysian MSM population to HIV-1 infection. In this context, educating this population on disease transmission and antiretroviral-based prevention strategy is imperative to reduce the magnitude of the epidemic. However, the effectiveness of this approach largely depends on factors such as ethnicity, race and the mindset of general population towards MSM population [2], that may decrease the health-seeking behaviours in MSM population [37]–[39]. In present study, no significant association was observed between clustering patterns with ethnicity, age and viral load.

Due to social stigmatization, discrimination and the underreported behaviour of MSM in population-based studies, we believe that the actual number of HIV-1 positive MSM in Kuala Lumpur could be higher than documented. Since the proviso in analyzing phylodynamic or evolutionary history more accurately relies significantly on the depth of population-based sampling, a study of such nature should be continued and expanded in order to improve the resolution of HIV-1 genomic diversity and transmission dynamics within the MSM networks.
